# The dynamics and efficacy of antiviral RNA silencing: A model study

**DOI:** 10.1186/1752-0509-2-28

**Published:** 2008-03-26

**Authors:** Marian AC Groenenboom, Paulien Hogeweg

**Affiliations:** 1 Theoretical Biology and Bioinformatics, University of Utrecht, Padualaan 8, 3584 CH Utrecht, The Netherlands

## Abstract

**Background:**

Mathematical modeling is important to provide insight in the complicated pathway of RNA silencing. RNA silencing is an RNA based mechanism that is widely used by eukaryotes to fight viruses, and to control gene expression.

**Results:**

We here present the first mathematical model that combines viral growth with RNA silencing. The model involves a plus-strand RNA virus that replicates through a double-strand RNA intermediate. The model of the RNA silencing pathway consists of cleavage of viral RNA into siRNA by Dicer, target cleavage of viral RNA via the RISC complex, and a secondary response. We found that, depending on the strength of the silencing response, different viral growth patterns can occur. Silencing can decrease viral growth, cause oscillations, or clear the virus completely. Our model can explain various observed phenomena, even when they seem contradictory at first: the diverse responses to the removal of RNA dependent RNA polymerase; different viral growth curves; and the great diversity in observed siRNA ratios.

**Conclusion:**

The model presented here is an important step in the understanding of the natural functioning of RNA silencing in viral infections.

## Background

RNA silencing is an evolutionary conserved regulation system and has an antiviral role in plants and some animals [1-3]. The key mediators of RNA silencing are small RNAs [4,5], that are cleaved from stem loop RNA or long stretches of double-strand RNA (dsRNA) by the enzyme Dicer [6,7]. The general view is that in antiviral silencing small interfering RNAs (siRNAs) are cleaved from long stretches of dsRNA, that are produced by the virus as intermediates in replication. The double-stranded siRNA associates with the protein complex RISC. The siRNA strand with the 5' lowest stability is selected to guide the RISC complex to the target. The siRNA-RISC complex cleaves the target and will stay intact to continue to the next target [8,9]. However, the long stretches of dsRNA that are formed during replication may not be accessible for Dicer [10], and recently it has been suggested that viral single-strand RNA (ssRNA) is cleaved into siRNAs [11].

In the primary silencing response siRNA is cleaved directly from viral RNA. In addition, in plants there can be a secondary response in which host encoded RNA dependent RNA polymerase (RDR) creates dsRNA that can be cleaved into one or more siRNAs.

We here present the first model study that combines viral growth with RNA silencing. Previous models focused either on virus dynamics or on RNA silencing separately, or on direct siRNA delivery for clinical applications of RNA silencing [12-16].

Previously we modeled the RNA silencing pathway and investigated the ability of RNA silencing to silence endogenous genes, transposons and dsRNA, and the role of the primary and secondary response in these cases [15]. Antiviral silencing differs from these processes, because the virus itself is replicating. We here model a replicating plus-strand RNA virus, and we extend our model of the silencing pathway with the kinetics of RISC, siRNA loaded RISC, Dicer and RDR, since we expect that the ability of the pathway to silence viruses will strongly depend on these proteins.

We study virus induced RNA silencing and its efficacy to reduce viral infections in a single compartment system. We find that RNA silencing can alter viral growth in five qualitatively different ways, and we compare the behavior in these regions to experimentally observed growth curves. We investigate the effect of different Dicer cleavage modes, and the impact of the secondary response. Lastly, we vary Dicer single- and double-strand cleavage rates and find that our model provides an explanation for the wide range of observed siRNA ratios.

## Methods

In short, the model consists of the following processes: The virus replicates itself through dsRNA, that is produced via virus encoded RNA dependent RNA polymerase (RdRP). Viral double- or single-strand RNA is degraded by host encoded Dicer into siRNAs, that have a plus- or minus-strand polarity. Via RISC the siRNAs cause degradation of either viral plus- or minus-strand RNA. In addition, siRNAs can be synthesized through the secondary pathway that involves synthesis of dsRNA by host encoded RDR. The model of the primary pathway can apply to both plants and animals, the model with secondary pathways applies to plants only [17].

We build up the mathematical model in a stepwise manner, starting with a model of viral replication. We then expand the model with the primary and finally the secondary silencing pathway. Each equation of the full model will consist of viral replication (V), the primary (P) and the secondary pathway. At the end of each equation we define V and P.

### Model description: Viral replication in a silencing defective system

**Figure 1:**
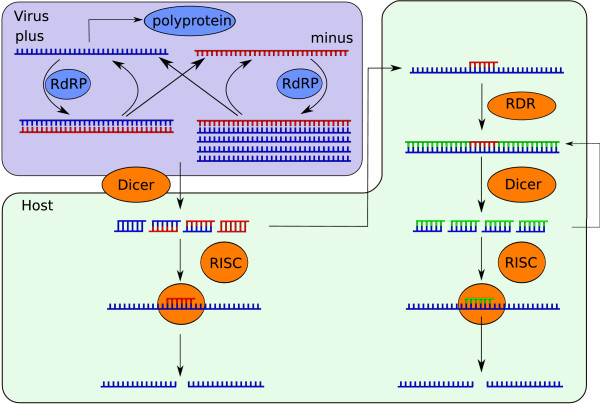
**Schematic representation of virus replication and antiviral silencing**. Schematic representation of plus-strand RNA virus replication (purple box) and the RNA silencing pathway (green box). Viral plus- and minus-strand RNA is replicated by RdRP, including semi conservative synthesis of multiple plus strands from a single minus-strand template. The formed dsRNA dissociates into single-strand RNA. Viral single- and double-stranded RNA can be cleaved by Dicer into siRNA. siRNA associates with RISC and cleaves the target RNA. siRNAs can guide or prime amplification of the response through host encoded RDR, or viral ssRNA is amplified in an unprimed manner. The primary pathway is shown on the left, the secondary on the right.

In plants the majority of viruses are plus-strand RNA viruses, that replicate via viral encoded RdRP. Our model of viral replication is based on the replication cycle shown in the box "Virus" in figure 1.

(1) $$\begin{array}{cc} \text{RdRP} & \frac{dR}{dt}=\left(\frac{rP}{(P+k_{t}}-d_{r}R+h(D_{p}+R_{a})-\{o(1-f)P+ofM+o_{d}D_{m}\}F\right)\equiv V_{r} \end{array}$$

(2) $$\begin{array}{cc} +\text{RNA} & \frac{dP}{dt}=\left(-o(1-f)PF+hD_{p}+hR_{a}-dP-\frac{vP^{5}}{k_{v}^{5}+P^{5}}\right)\equiv V_{p} \end{array}$$

(3) $$\begin{array}{cc} -\text{RNA} & \frac{dM}{dt}=\left(-ofMF+hD_{p}-dM+hD_{m}{\left(1-\frac{1}{D_{m}}\right)}^{\left(R_{a}-D_{m}\right)}\right)\equiv V_{m} \end{array}$$

(4) $$\begin{array}{cc} \text{Virions} & \frac{dV}{dt}=\frac{vP^{5}}{k_{v}^{5}+P^{5}}\equiv V_{v} \end{array}$$

(5) $$\begin{array}{cc} \text{dsRNA} & \frac{dD_{p}}{dt}=o(1-f)PF-hD_{p}\equiv V_{dp} \end{array}$$

(6) $$\begin{array}{cc} \text{dsRNA} & \frac{dD_{m}}{dt}=ofMF-hD_{m}{\left(1-\frac{1}{D_{m}}\right)}^{\left(R_{a}-D_{m}\right)}\equiv V_{dm} \end{array}$$

(7) $$\begin{array}{cc} \text{act}\text{.RdRP} & \frac{dR_{a}}{dt}=ofMF+o_{d}D_{m}F-hR_{a}\equiv V_{ra} \end{array}$$

As indicated *R*, *P*, *M *and *V *represent the number of viral RdRP molecules, plus-strand RNAs, minus-strand RNAs and virions, respectively. *D*_*p*_, *D*_*m *_and *R*_*a *_represent the number of dsRNA complexes produced from the plus-strand, dsRNA complexes from the minus-strand and the total number of RdRPs producing plus-strand RNA from a minus-strand template, respectively.

RdRP is not packaged in the virions, therefore the first step in replication is the translation of RdRP from the viral plus strand with maximum rate *r *and saturation constant *k*_*t *_. RdRP associates with plus- or minus-stranded RNA with maximum rate *o *to synthesize a complementary strand. *f *sets the preference of RdRP for the minus strand. We assume that the minus-strand is the preferred template for dsRNA synthesis. The complex formation (F) between RdRP and RNA strands is saturated for both viral RNA and RdRP (the Beddington-DeAngelis functional response [18,19]):

(8) $$F=\frac{R}{R+P+M+D_{m}+k_{r}}$$

with saturation constant *k*_*r*_. Semi-conservative synthesis of multiple plus-strands from a single minus-strand template is incorporated in the model. RdRP can associate with *D*_*m *_(dsRNA complexes formed from the minus strand) with maximum rate *o*_*d*_. In the case of *D*_*p *_a single RdRP is present on the complex, and the entire complex dissociates with rate *h *into RdRP and a plus- and minus-strand. Multiple RdRPs are present on *D*_*m*_, and each RdRP produces one product strand and then dissociates with rate *h*. However, the complex still exists after dissociation of one RdRP. The *D*_*m *_complex disappears when the last RdRP dissociates. The chance that only one RdRP is present is ${\left(1-\frac{1}{D_{m}}\right)}^{\left(R_{a}-D_{m}\right)}$ and therefore the dissociation rate of *D*_*m *_is $hD_{m}{\left(1-\frac{1}{D_{m}}\right)}^{\left(R_{a}-D_{m}\right)}$.

The virus produces virions that consist of plus-strand RNA and coat proteins. We simplify here by using the number of plus-stranded RNA instead of modeling a separate coat protein. The virion production has a maximum rate of virion production *v *and a Michaelis Menten constant *k*_*v*_. *d *and *d*_*r *_are the decay rates of viral ssRNA and RdRP respectively.

### Model description: antiviral RNA silencing

#### Primary response

The viral replication model is extended to include viral induction of the RNA silencing pathway. The scheme is shown in Figure 1. The V terms represent the parts of the equations modeling viral replication, and are defined in Equation 1–7.

(9) $$\begin{array}{cc} \text{RdRP} & \frac{dR}{dt}=V_{r}+G_{d}(D_{p}+R_{a})\equiv V_{r}+{\text{P}}_{r} \end{array}$$

(10) $$\begin{array}{cc} +\text{RNA} & \frac{dP}{dt}=V_{p}-\frac{b_{2}R_{m}P}{P+k_{ri}}-G_{s}P\equiv V_{p}-{\text{P}}_{p} \end{array}$$

(11) $$\begin{array}{cc} -\text{RNA} & \frac{dM}{dt}=V_{m}-\frac{b_{2}R_{p}M}{M+k_{ri}}-G_{s}M\equiv V_{m}+{\text{P}}_{m} \end{array}$$

(12) $$\begin{array}{cc} \text{dsRNA} & \frac{dD_{p}}{dt}=V_{dp}-G_{d}D_{p}\equiv V_{dp}+{\text{P}}_{dp} \end{array}$$

(13) $$\begin{array}{cc} \text{dsRNA} & \frac{dD_{m}}{dt}=V_{dm}-G_{d}D_{m}\equiv V_{dm}+{\text{P}}_{dm} \end{array}$$

(14) $$\begin{array}{cc} \text{act}\text{.RdRP} & \frac{dR_{a}}{dt}=V_{ra}-G_{d}R_{a}\equiv V_{ra}+{\text{P}}_{ra} \end{array}$$

(15) $$\begin{array}{cc} +\text{siRNA} & \frac{dSi_{p}}{dt}=\left(G_{s}P+0.5G_{d}(D_{p}+D_{m})-d_{si}Si_{p}-b_{1}Si_{p}R_{f}\right)\equiv {\text{P}}_{sip} \end{array}$$

(16) $$\begin{array}{cc} \text{free RISC} & \frac{dR_{f}}{dt}=i-d_{r}R_{f}-b_{1}R_{f}\left(Si_{p}+Si_{m}\right)\equiv {\text{P}}_{rf} \end{array}$$

(17) $$\begin{array}{cc} +\text{RISC} & \frac{dR_{p}}{dt}=b_{1}R_{f}Si_{p}-d_{r}R_{p}\equiv {\text{P}}_{rp} \end{array}$$

Variables *Si*_*p*_, *Si*_*m*_, *R*_*f*_, *R*_*p *_and *R*_*m *_represent siRNA cleaved from plus-strands, siRNA from minus-strands, free RISC, RISC loaded with *Si*_*p*_, and RISC loaded with *Si*_*m*_, respectively. We do not show the equations for *Si*_*m *_and *R*_*m *_since they have the same form as the equations for *Si*_*p *_and *R*_*p*_. The equation for the virions is unaltered (eq. 4).

Viral RNA is cleaved into siRNAs by Dicer. We simplify here by using only a single type of Dicer. $G_{d}$ And $G_{s}$ are the Dicer cleavage functions for dsRNA and ssRNA respectively.

(18) $$\begin{array}{cc} G_{d}=\frac{c_{d}D_{i}}{D_{p}+D_{m}+k_{d}} & G_{s}=\frac{c_{s}D_{i}}{P+M+k_{d}} \end{array}$$

*D*_*i *_is the number of Dicer molecules present in the host. Dicer can cleave siRNAs from dsRNA with maximum rate *c*_*d*_, and from ssRNA with rate *c*_*s*_. When Dicer cleaves both single- and double-strand RNA, we use the following function:

(19) $$G_{d,s}=\frac{c_{d,s}D_{i}}{D_{p}+D_{m}+P+M+k_{d}}$$

that is saturated for all possible Dicer targets. When Dicer cleaves dsRNA a 1-1 ratio of siRNAs targeting the plus- and minus-strand is produced. When studying the effects of Dicer cleavage rate, we always use the total rate, that is, *c*_*d *_+ 2*c*_*s*_.

siRNAs associate with rate *b*_1 _with RISC to form siRISC: the active RISC complex that will cause the breakdown of viral RNA. One strand of the siRNA is kept in siRISC, and since that strand consists of a short stretch of either plus- or minus-strand viral RNA, it will match to the complementary strand. The target of the active RISC is the RNA strand that matches the incorporated siRNA. siRISC cleaves the target RNA with maximum rate *b*_2_. *k*_*ri *_is the saturation constant of the siRISC cleavage function. *d*_*si *_and *d*_*r *_are the siRNA and RISC decay rates.

#### Secondary response

In addition to the primary response, the silencing pathway can include a secondary response. Host-encoded RDR synthesizes dsRNA from single-strand substrates, that are cleaved into secondary siRNAs. In vitro RDR has two modes of action: primed and unprimed amplification [20,21]. In the case of primed amplification siRNA binds to ssRNA and serves as a primer for RDR. In the case of unprimed amplification, RDR synthesizes dsRNA from ssRNA without a primer. Recently, a third possibility has been proposed: RDR is guided by a siRNA to the ssRNA after which unprimed amplification takes place [22,23]. The model is expanded with the three amplification terms:

(20) $$A_{u}=\frac{a_{u}}{\left(P+M+k_{a}\right)}$$

(21) $$A_{g}=A_{p}=\frac{a_{p,g}}{\left((Si_{p}+Sis_{p})M+(Si_{m}+Sis_{m})P+k_{a}\right)}$$

Where $A_{u}$ is unprimed amplification, $A_{p}$ is primed amplification and $A_{g}$ is guided amplification. We study the amplification pathways separately. In the case of guided amplification, the siRNAs are not removed when they guide amplification, in contrast to primed amplification. Amplification produces dsRNA that is not used for virus replication (*D*_*e*_). This dsRNA is degraded into secondary siRNAs with a plus- or minus-strand polarity; *Sis*_*p *_and *Sis*_*m *_respectively. Since Dicer now cleaves *D*_*e *_in addition to *D*_*p *_and *D*_*m*_, the Dicer cleavage functions $G_{d}$ and $G_{d,s}$ are saturated for *D*_*e*_:

(22) $$G_{d}=\frac{c_{d}D_{i}}{D_{p}+D_{m}+D_{e}+k_{d}}$$

(23) $$G_{d,s}=\frac{c_{d,s}D_{i}}{D_{p}+D_{m}+D_{e}+P+M+k_{d}}$$

V and P represent the parts of the equations that describe virus replication and the primary silencing pathway, and are defined in Equation 1–7 and Equation 9–17. The equations altered by the secondary response are:

(24) $$\begin{array}{cc} +\text{RNA} & \frac{dP}{dt}=V_{p}-{\text{P}}_{p}-\frac{b_{2}R_{sm}P}{P+k_{ri}}-A_{u}P\\ & -A_{p}(Si_{m}+Sis_{m})P\\ & -A_{g}(Si_{m}+Sis_{m})P \end{array}$$

(25) $$\begin{array}{cc} -\text{RNA} & \frac{dM}{dt}=V_{m}-{\text{P}}_{m}-\frac{b_{2}R_{sp}M}{P+k_{ri}}-A_{u}M\\ & -A_{p}(Si_{p}+Sis_{p})M\\ & -A_{g}(Si_{p}+Sis_{p})M \end{array}$$

(26) $$\begin{array}{cc} +\text{siRNA} & \frac{dSi_{p}}{dt}={\text{P}}_{sip}-A_{p}Si_{p}M \end{array}$$

(27) $$\begin{array}{cc} \sec.D & \frac{dD_{e}}{dt}=A_{p}((Si_{m}+Sis_{m})P+(Si_{p}+Sis_{p})M)\\ & -A_{g}((Si_{m}+Sis_{m})P+(Si_{p}+Sis_{p})M)\\ & -A_{u}(P+M)-G_{d}D_{e} \end{array}$$

(28) $$\begin{array}{ll} \sec.Si_{p} & \frac{dSis_{p}}{dt}=0.5G_{d}D_{e}-d_{si}Sis_{p}-b_{1}Sis_{p}R_{f}\\ & -A_{p}Sis_{p}M \end{array}$$

(29) $$\begin{array}{cc} \sec.\text{RISC} & \frac{dR_{sp}}{dt}=b_{1}Sis_{p}R_{f}-d_{r}R_{sp} \end{array}$$

With *D*_*e*_, *Sis*_*p*_, *Sis*_*m*_, *R*_*sp *_and *R*_*sm *_representing dsRNA produced via amplification, secondary siRNA with plus strand polarity, secondary siRNA with minus strand polarity, RISC loaded with *Sis*_*p *_and RISC loaded with *Sis*_*m*_, respectively. Again we do not show *Si*_*m*_, *Sis*_*m *_and *R*_*m *_since they have the same form as *Si*_*p*_, *Sis*_*p *_and *R*_*p *_respectively. The full model can be found in the Appendix.

### Parameters

Where possible, parameters were taken from literature (Table 1). We have estimated the remaining parameters within reasonable ranges as indicated in Table 1. When choosing parameters we aimed to show all qualitative outcomes of the model. When more parameters of a specific case are known, we can fit the model to that case, and investigate what behavior is expected.

**Table 1:** Parameters used in the model with their default value and the studied range. #mol is number of molecules.

**Par**.	**Meaning**	**Value units**	**Studied range**	**Reference**
*r*	maximum translation rate * #ribosomes	15*5000 #mol hr^-1^	30,000 – 750,000	[38,39]
*o*	max rate of complex formation ssRNA	1 hr^-1^	0.1 – 5	
*o*_*d*_	max rate of complex formation dsRNA	100 hr^-1^	0 – 1000	
*f*	ratio of binding plus or minus RNA	0.9 -	0 – 1	
*h*	dsRNA-RDR splitting rate	10 hr^-1^	1 – 1,000	
*v*	max virion production rate	500 #mol hr^-1^	0 – 50,000	

*D*_*i*_	number of Dicer molecules	500 #mol	0 – 5,000	
*c*_*d*_	max Dicer cleavage rate for dsRNA	3 #mol hr^-1^	0 – 20	
*c*_*s*_	max Dicer cleavage rate for ssRNA	3 #mol hr^-1^	0 – 20	
*b*_1_	rate of RISC activation	0.005 #mol ^-1 ^hr^-1^	0 – 1	
*b*_2_	RISC target cleavage rate	20 #mol ^-1 ^hr^-1^	0 – 1,000	[8]
*i*	translation of RISC	100 #mol hr^-1^	0 – 1,000	
*a*	amplification (*a*_*u*_, *a*_*p *_and *a*_*g*_)	100 #mol hr^-1^	0 – 400	

*d*_*r*_	decay RDR and RISC	0.1 hr^-1^	0 – 0.5	
*d*	decay viral ssRNA	0.5 hr^-1^	0 – 2	
*d*_*si*_	decay siRNA	2 hr^-1^	0 – 5	[40]

*k*_*v*_	saturation of virion production	10,000 #mol	1 – 100,000	
*k*_*d*_	saturation of Dicer cleavage	10,000 #mol	1 – 100,000	
*k*_*t*_	saturation constant for translation	1,000 #mol	1 – 10,000	
*k*_*ri*_	saturation of RISC cleavage	1,000 #mol	1 – 10,000	
*k*_*r*_	saturation of complex formation	1,000 #mol	1 – 10,000	
*k*_*a*_	saturation amplification	1000 #mol	1 – 10,000	

## Results and Discussion

### Viral growth in a silencing defective system

We first study the growth of a plus-strand RNA virus in a silencing defective system. The viral replication cycle is shown in Figure 1, and the model description can be found in the Methods.

**Figure 2:**
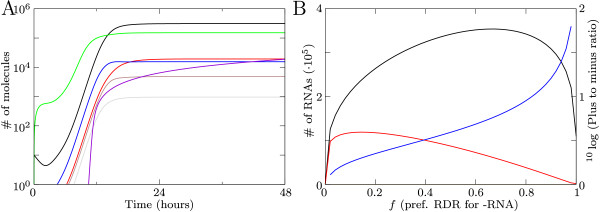
**Viral growth in a silencing deficient system**. (A) Timeseries of virus growth showing the number plus-strand RNA (black); minus-strand RNA (red); free RDR (green); dsRNA complex from plus strand (gray), dsRNA complex from minus strand (brown); RDRs active on minus strands (blue); and the number of virions (violet). (B) The effect of *f *on the number of plus and minus strands, with plus-strand RNA (black) and minus-strand RNA (red). The blue line shows the effect on the plus-to-minus strand ratio.

In the default setting our model results in a sigmoid growth of viral plus-strands (Figure 2a). Sigmoid growth curves for plus-strand RNA viruses in silencing defective systems have been observed for example for West Nile virus [24], Japanese encephalitis virus [25] and vesicular stomatitis virus in *C. elegans *[26]. In our model, after initialization with 10 viral plus-strands, the number of free plus-strands initially decreases, because they become part of the RdRP-RNA complex. After this decrease, the virus grows exponentially and then growth saturates due to the saturation in the production of RDR and in the dsRNA complex formation. In the equilibrium the number of plus-strand RNA is an order of magnitude higher than the number of minus strands.

Depending on the initial dose and growth parameters, the virus can expand to the equilibrium as shown in Figure 2a, or it dies out immediately. An increase in *o*, *o*_*d*_, *h *and *r *causes faster initial growth of the virus. A decrease in *k*_*r *_and *k*_*t *_has the same effect. A sigmoid curve with a higher equilibrium can be obtained by increasing *o*, *o*_*d *_and *r*, and by decreasing *h*. Changing *v *does not have a large effect on viral growth, except when the timing of virion production is too early (when *k*_*v *_is low).

The plus-to-minus ratio is controlled by *f*, the preference that RDR has for minus-strands. When increasing *f *the plus-to-minus ratio becomes increasingly biased towards the plus strand (Figure 2b). The virus is only capable of expanding when *f *lies between 0.009 and 0.988, and the ratio of plus-to-minus strands can vary from less then 1 up to 100. Parameter *o*_*d *_can also increase the plus-to-minus ratio: when more RdRPs are able to bind to minus RNA, the ratio can become more skewed. However, the dsRNA complex has to stay intact long enough to observe this effect. By varying the above parameters we can adjust the viral growth curve to fit different viruses.

### Virus induced silencing

#### Primary response

We add the host primary RNA silencing defense to the viral growth model. Dicer cleaves dsRNA or ssRNA into siRNAs, that associate with free RISC and will target either the plus- or minus-strand. The primary RNA silencing pathway can alter the viral growth pattern substantially: it can slow down and decrease viral growth, cause oscillations, or can result in complete virus clearance.

The important silencing parameters are the RISC activation rate (*b*_1_), the RISC cleavage rate (*b*_2_), the number of Dicers (*D*_*i*_), the influx of free RISC (*i*), and the siRNA production rate (*c*_*d *_and *c*_*s*_). All these parameters determine the rate at which RNA silencing degrades viral RNA. We refer to this effect as the silencing strength. We will use one parameter as an example to study the effect of silencing strength.

As the functioning of Dicer in virus derived siRNAs is not resolved yet, we investigate three different Dicer activity modes: Dicer can cleave either only dsRNA, only ssRNA or both.

#### Dicer on dsRNA

In this setting Dicer cleaves only dsRNA and therefore siRNAs targeting the plus- and minus-strand are always present in equal amounts.

**Figure 3:**
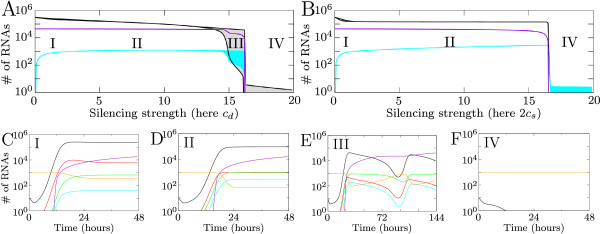
**Effect of RNA silencing on viral growth**. Four qualitative different outcomes of viral growth and silencing. (A) and (B) Bifurcation diagrams for increasing silencing strength. For varying silencing strength the number of plus-strand RNAs 100 hours post infection is shown. Black lines indicate plus-strand RNA, violet lines the number of virions produced, and cyan lines the total number of siRNAs. In the filled area a single peak or multiple oscillations of the number of plus-strand RNAs or the number of siRNAs occur. (A) Dicer cleaves dsRNA and (B) Dicer cleaves ssRNA. (C)-(F) show typical timeseries of behavior in the four regions for *c*_*d *_= 2, *c*_*d *_= 6, *c*_*d *_= 8 and *c*_*d *_= 10. Timeplots show plus-strand RNA (black); minus-strand RNA (red); total number of virions (violet); free siRNA (cyan); free RISC (orange); active RISC (green). Virions can only be formed when virus levels are sufficiently high.

To investigate the effect that RNA silencing has on viral growth we vary the silencing strength. We do this by varying any of the silencing parameters according to the ranges in Table 1. As an example we here use the siRNA production rate *c*_*d *_by Dicer. For each value of *c*_*d *_we plot the number of viral plus-strand RNA, siRNA and virions present 100 hours (4.2 days) post infection. Maximum and minimum values are monitored from 10 hours onwards. When silencing strength increases, we observe four possible effects of silencing. The four regions of different behavior are shown in the bifurcation diagram in Figure 3a. In Figures 3c–f timeseries of the behavior in the different regions are shown.

For a low silencing strength, in region I, virus levels are slightly decreased. A timeseries of this behavior is shown in Figure 3c. A small peak in virus levels occurs, after which a decreased equilibrium amount of RNA is reached. When silencing strength increases, viral growth is delayed and the equilibrium is decreased (Region II). The delayed virus growth results in a later timing and thereby decrease of virion production.

A further increase in silencing strength leads to the behavior in region III, where oscillations in viral RNA levels occur (gray area in Figure 3a, and timeplot in Figure 3e). The oscillations are caused by the feedback of the system: the silencing response needs the virus to be sustained, but at the same time eliminates it. When silencing decreases viral RNA substantially, less siRNAs can be produced and the weakening silencing response creates the opportunity for the virus to expand again. Each time when the number of plus strands increases, more virions are produced, resulting in a stepwise virion production.

For even higher silencing strength the virus is not able to expand and decreases directly after initiation (Region IV, Figure 3f). In this case the virus is not able to produce virions.

The silencing parameters are determined by the host, but the silencing strength is also determined by properties of the virus. Folding and accessibility of viral RNA can limit the amount of RNA accessible for Dicer. Additionally, the RISC cleavage rate depends on target accessibility [27]. Therefore, the virus itself affects silencing strength and the bifurcation diagram can be interpreted in terms of viral properties. In Figure 3a a low silencing strength corresponds with a very resistant virus and a high silencing strength with a weaker virus. Therefore, it is possible that a single host type-with fixed silencing parameters- is able to silence one virus, but not the other.

#### Dicer on ssRNA, and on both ds and ssRNA

Next we assume that Dicer cleaves only ssRNA and has equal affinity for plus- and minus-strands (Figure 3b). There are some striking differences between the case where Dicer cleaves long dsRNA and this case. A major difference is that oscillations do not occur, instead region IV follows directly after region II. Additionally, in region II an increase in silencing strength does not affect the amount of virus present, while for dsRNA cleavage by Dicer the amount of virus slopes downward due to increasing silencing strength.

**Figure 4:**
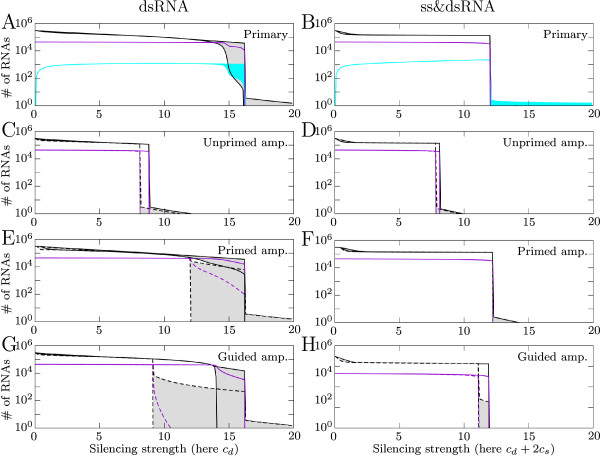
**Effect of amplification**. Bifurcation diagrams for increasing silencing strength. In this case we varied Dicer cleavage rates. On the left, Dicer cleaves dsRNA; on the right Dicer cleaves dsRNA and plus- and minus-strand ssRNA. In the case of Dicer cleaving plus- and minus-strand RNA *c*_*d *_= *c*_*s*_. Solid black lines show the number of plus-strand RNA 300 hours post infection, violet lines show the number of virions, and cyan lines the total number of siRNAs. Dashed lines show the behavior when amplification yields four siRNAs per amplified transcript. For clarity the lines representing siRNAs have been omitted in figure C-H.

When single and double-stranded RNA cleavage by Dicer are combined, the Dicer cleavage function is saturated for both single- and double-strand RNA. Interestingly, a combined single- and double-strand cleavage by Dicer makes the response more efficient than each case separately (figure 4b).

#### Secondary response

An important component of the silencing pathway is amplification via RDR. Detailed information on RDRs can be found in Wassenegger and Krczal [28]. RDR -and therefore amplification- has been shown to play an antiviral role in plants, and in RDR knockouts virus accumulated to higher levels and lead to increased symptom severity [29-34]. Yu et al. [31] have shown that in an RDR defective mutant viral RNA turnover is substantially decreased compared to the wild-type. Several experiments have shown that RDR plays a role in the systemic spread of the virus to the plant meristem and newly emerging leaves [32-34].

We studied the effect that the secondary response can have. We implement all three types of amplification: primed, unprimed and guided amplification. For each type of amplification we investigate two different Dicer activity modes. Either Dicer can cleave siRNAs from dsRNA only, or Dicer can cleave both dsRNA and ssRNA. The case where Dicer cleaves only ssRNA is not relevant here because the amplification functions through dsRNA.

Again we vary the silencing strength by varying *c*_*d *_and *c*_*s*_. As previously we can interpret the results also in terms of viral properties. The addition of the secondary response can have a number of effects. The virus can be cleared for lower silencing strength, and oscillations can be enlarged (Figure 4). The most striking is the occurrence of a new region of behavior, where the virus is able to expand initially but is cleared later. The different types of amplification have different effects.

#### Unprimed amplification

The solid lines in Figure 4c and 4d show the bifurcation diagram when the gain of unprimed amplification is one siRNA per synthesized RDR transcript. Silencing becomes effective for lower silencing strength when unprimed amplification is added. This means that viruses that could not be silenced with the primary response can be silenced after the addition of the secondary response. When the gain is increased to four siRNAs, there is hardly an effect (dashed lines Figure 4c), indicating that maximum efficiency is already reached when the gain is only one siRNA.

Interestingly, the oscillations that occurred without amplification have disappeared. When the amplification rate is lower, oscillations occur in the same region as without amplification. With increasing amplification rate the transition to region IV moves to lower silencing strength, cutting off the bifurcation diagram and losing the oscillatory regime.

#### Primed amplification

**Figure 5:**
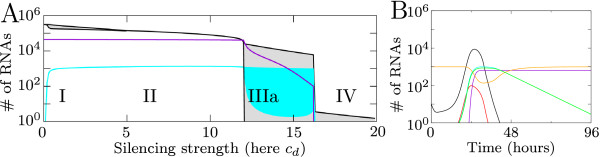
**Behavior in region IIIa**. (A) Region IIIa shown in a bifurcation diagram for increasing Dicer cleavage rate (*a*_*p *_= 100, 4 siRNAs per amplified transcript). Black lines indicate plus-strand RNA, violet lines the number of virions produced, and cyan lines the total number of siRNAs. (B) A timeplot showing the behavior in region IIIa (*c*_*d *_= 15) with plus-strand RNA (black); minus-strand RNA (red); total number of virions (violet); total siRNA (cyan); free RISC (orange); active RISC (green).

At first primed amplification seems to have little effect on silencing (solid lines, Figure 4e and 4f). This is caused by the lack of gain by this amplification route. Primed amplification uses one siRNA to produce a siRNA, resulting in a gain of zero. When four siRNAs are cleaved from an amplified transcript (*D*_*e*_), there is a clear effect (dashed lines, Figure 4e). The oscillations in region III are enlarged to such extent that the virus is cleared after an initial growth peak. This is a new region of behavior, region IIIa (Figure 5). In this region the virus can only produce virions during the initial growth peak, resulting in a strongly decreased virion production. Region IIIa can also occur without amplification for a faster growing virus. When the initial growth peak is sufficiently high and silencing is strong, the virus can be cleared after a single peak.

When Dicer cleaves both single- and double-strand RNA, primed amplification has virtually no effect, indicating that sufficient dsRNA cleavage is necessary to benefit from primed amplification.

#### Guided amplification

Guided amplification does not deplete the siRNAs as primed amplification does. In the oscillatory regime, the oscillations are enlarged and, as with primed amplification, region IIIa behavior is observed (Figure 4g). When Dicer cleaves single- and double-strand RNA this region occurs only when the gain of amplification is higher (Figure 4h).

#### The role of the secondary response

We conclude that enough dsRNA cleavage is necessary to observe an effect of the secondary pathway. Not all virus infections seem to be affected by the secondary response. RDR defective Tobacco (*N. bentamiana*) plants, that are not capable of a secondary response, were only hypersusceptible to some of the tested viruses, while the response to other viruses was not affected [32]. Although these observations seem contradictory, our model suggests that it is an inherent property of the system that, depending on the virus, RDR can or cannot influence the amount of virus accumulation. The initial position of the system in the bifurcation diagram influences the effect of RDR. Depending on the initial position of the system, the removal of RDR can bring the system from region I to II, IIIa to II or III, IV to II or III, or it remains in the same region of behavior. This means that viral properties such as accessibility of viral single and double-strand RNA to Dicer, availability of ssRNA to RISC, and viral growth rate define the effect that RDR can have.

### Observed ratio of siRNAs

Several studies have focused on determining the ratio between plus- and minus-strand derived siRNAs during viral infection. Molnar et al. [11] studied the siRNA origin of *Cymbidium ringspot tombusvirus *in detail. They found that 80% of the siRNAs were derived from the plus-strand. Additionally, they studied *Tobacco mosaic virus *and *Potato virus X*, and found that these siRNA ratios were also biased towards the plus-strand. They suggested that the presence of more plus-strand derived siRNAs increases silencing efficiency, and that Dicer may have a preference for plus-strand RNA. It may be more efficient to mainly target the minus-strand, because it is present in lower amounts. Pantaleo et al. [9] confirmed the skewed siRNA ratio found by Molnar et al. [11], but reported that the asymmetry was not as pronounced. Ho et al. [35] explored two other viruses and found that for *Turnip crinkle carmovirus *97.6% of the siRNAs was derived from the plus strand, while for *Turnip mosaic potyvirus *only 58.1% was produced from the plus-strand. The question rises if these different ratios can arise without implementing Dicer preferences for the plus strand.

**Figure 6:**
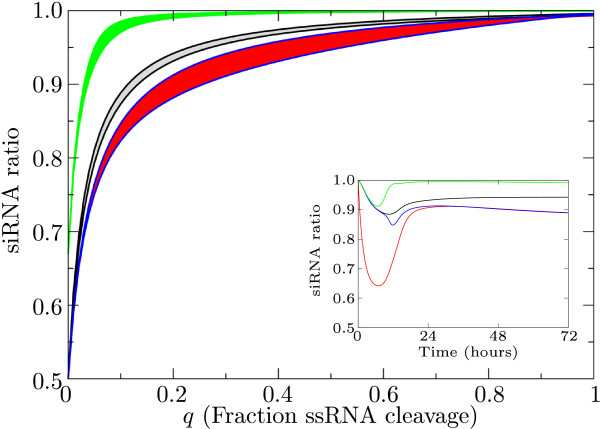
**siRNA ratios**. siRNA ratios observed for varying Dicer cleavage modes: on the left Dicer cleaves exclusively dsRNA; on the right exclusively ssRNA. Shown are siRNA ratios for the primary pathway (black); primed amplification (green), unprimed amplification (red) and guided amplification (blue). Unprimed and guided amplification result in the same siRNA ratios, the lines lie on top of each other. The inset shows the change in siRNA ratio in time for all studied pathways (fraction of ssRNA cleavage *q *is 0.2).

To investigate whether a preference of Dicer is needed to explain the observed ratios we vary the ratio between single- and double-stranded RNA cleavage by Dicer (*q*). This ratio may be affected by viral properties as RNA folding and accessibility of single- and double-strand RNA. We keep total Dicer cleavage rate constant and plot the minimum and maximum siRNA ratio observed between 24 and 100 hours post infection (Figure 6). The cleavage function is saturated according to the ratio between single- and double-stranded RNA cleavage by Dicer:

(30) $$G_{d}=\frac{(1-q)c_{d}D_{i}}{(1-q)(D_{p}+D_{m}+D_{e})+q(P+M)+k_{d}}$$

(31) $$G_{s}=\frac{qc_{s}D_{i}}{(1-q)(D_{p}+D_{m}+D_{e})+q(P+M)+k_{d}}$$

We set total Dicer cleavage rate to region I or II (total Dicer cleavage rate is 3) to avoid non-existent siRNA ratio's in region III, IIIa or IV.

When only dsRNA is cleaved, the siRNA ratio will be 0.5, due to the fact that there is always an 0.5 chance that either siRNA strand is selected. The addition of primed amplification can raise this ratio: more minus-strand siRNAs are used for amplification because the plus strand is present in higher amounts. When only ssRNA is cleaved, the ratio follows from the plus-to-minus ratio produced by the virus. We find that when Dicer cleavage rates are intermediate between these two extreme cases, different siRNA ratios can occur for different viruses. In fact, the whole range between 0.5 and 1 is possible, and the experimentally observed ratios can be reached without implementing an extra mechanism or Dicer preference for the plus-strand. When the virus ssRNA has many hairpins and mainly ssRNA is cleaved, we predict a high siRNA ratio. When mainly dsRNA is cleaved we expect a low ratio (Figure 6).

The siRNA ratios are shown in ranges between the maximum and minimum because the siRNA ratio changes over time. The trend is that initial siRNA ratio's are close to 1 (almost only plus-strand derived), followed by a decline in siRNA ratio, after which a stable ratio is reached (Figure 6).

Concluding, when Dicer is capable of both single- and double-strand RNA cleavage, the experimentally observed ratios can be reached without implementing a Dicer preference for the plus-strand.

## Conclusion

The RNA silencing pathway functions as an immune system in plants and several animals. We here presented the first model study that combines viral growth and RNA silencing.

In contrast to the previously reported necessity of a nonlinear feedback in amplification to silencing transgenes [15], we showed that antiviral silencing can function without any amplification. In agreement with our results, experiments have shown that plants with a defective RDR are still capable of antiviral silencing [36].

RNA silencing can alter the sigmoid growth pattern of the virus substantially. Wilkins et al. [26] studied viral growth in *C. elegans *with silencing able and silencing deficient hosts. They show that without silencing the virus grows according to a sigmoid curve to 1 · 10^6^, with silencing this curve is flattened and the virus accumulates to 5 · 10^4^. This behavior is in accordance with our results: in region I and II the virus equilibrium is decreased to similar extent. Dzianott and Bujarski [37] obtained a *Brome mosaic virus *growth curve in *A. thaliana*. They observed an initial decline in RNA, then a peak of viral growth, after which the concentrations decline toward zero again. We can assume the curve found by Dzianott and Bujarski [37] represents viral growth limited by RNA silencing, because siRNA was found, and virus accumulated to higher levels in a host expressing a silencing suppression protein. This 'peak' behavior corresponds with the behavior of our model in region IIIa. Region IIIa occurs only when the virus and the silencing response are fast, with amplification these conditions are more likely to occur.

We studied three cases of Dicer activity modes: Dicer can cleave dsRNA, ssRNA or both. We show that a combined degradation of single- and double-strand RNA by Dicer is more efficient than each case separately.

Sufficient dsRNA cleavage is necessary to observe an effect of the secondary silencing response. Unprimed amplification is able to strongly increase the efficacy of RNA silencing, clearing the virus for much lower silencing strength. Virion production is then not possible, because virus growth is completely silenced. Primed and guided amplification can cause major oscillations that also lead to clearance of the virus, however, the virus is still able to produce virions during the initial growth peak. Moreover, primed amplification can only be beneficial when the siRNA gain through amplification is sufficiently high.

Experiments have shown that viruses accumulate to higher levels in hosts defective in the secondary response [29-34]. However, some viruses are and some viruses are not limited by RDR (secondary response) [32]. Our model study has shown that such seemingly contradictory results can be explained by a slight change in viral properties that bring the system to a different region of behavior.

In antiviral silencing a wide range of siRNA ratios has been observed [9,11,35]. Our model provides a possible explanation for these observations. Each virus has unique folding properties and accessibility of its RNA, thereby affecting the Dicer rate on single- and double-strand RNA. Variation in these Dicer cleavage rates can account for the full range of observed siRNA ratios and a preference of Dicer for either the plus- or minus-strand is not needed. The model presented here studies antiviral silencing on the cellular level, and is a first step in understanding the interactions between viruses and RNA silencing. An interesting extension of the model is the addition of virus encoded silencing suppressors. Although we expect that silencing suppression partly overlaps with decreasing silencing strength, the specific types of silencing suppressors could affect the RNA silencing response in unexpected ways. Additionally we plan to extend our model with the spread of virus particles and siRNAs from cell to cell. We hope that further experimental research will be done to provide more data to test our model. We would be specifically interested in timeseries of viral growth with and without RNA silencing, and in particular in the role of RDR and Dicers on the dynamics of RNA silencing within the cell.

Our work provides an important framework to study natural antiviral silencing. We have shown that various experimentally observed behaviors can be explained, even when they seem contradictory at first.

## Authors' contributions

MG and PH conceived and designed the models, and wrote the paper. MG performed the numerical computations.

## Appendix

The full model:

$$\begin{array}{c} \frac{dR}{dt}=\frac{rP}{\left(P+k_{t}\right)}-d_{r}R-\{o(1-f)P+ofM+o_{d}D_{m}\}F\\ +hD_{p}+hR_{a}+G_{d}(D_{p}+R_{a})\\ \frac{dP}{dt}=-o(1-f)PF+hD_{p}+hR_{a}-dP-\frac{vP^{5}}{k_{v}^{5}+P^{5}}\\ -\frac{b_{2}R_{m}P}{P+k_{ri}}-G_{p,m}P-\frac{b_{2}R_{sm}P}{P+k_{ri}}\\ -A_{u}P-A_{p}(Si_{m}+Sis_{m})P-A_{g}(Si_{m}+Sis_{m})P\\ \frac{dM}{dt}=-ofMF+hD_{p}+hD_{m}{\left(1-\frac{1}{D_{m}}\right)}^{\left(R_{a}-D_{m}\right)}-dM\\ -\frac{b_{2}R_{p}M}{M+k_{ri}}-G_{p,m}M-\frac{b_{2}R_{sp}M}{P+k_{ri}}-A_{u}M\\ -A_{p}(Si_{p}+Sis_{p})M-A_{g}(Si_{p}+Sis_{p})M\\ \frac{dV}{dt}=\frac{vP^{5}}{k_{v}^{5}+P^{5}}\\ \frac{dD_{p}}{dt}=o(1-f)PF-hD_{p}-G_{d}D_{p}\\ \frac{dD_{m}}{dt}=ofMF-hD_{m}{\left(1-\frac{1}{D_{m}}\right)}^{\left(R_{a}-D_{m}\right)}-G_{d}D_{m}\\ \frac{dR_{a}}{dt}=ofMF+o_{d}D_{m}F-hR_{a}-G_{d}R_{a}\\ \frac{dSi_{p}}{dt}=G_{p,m}P+0.5G_{d}(D_{p}+D_{m})-d_{si}Si_{p}-b_{1}Si_{p}R_{f}\\ -A_{p}Si_{p}M\\ \frac{dSi_{m}}{dt}=G_{p,m}M+0.5G_{d}(D_{p}+D_{m})-d_{si}Si_{m}-b_{1}Si_{m}R_{f}\\ -A_{p}Si_{m}P\\ \frac{dR_{f}}{dt}=i-d_{r}R_{f}-b_{1}R_{f}(Si_{p}+Si_{m})\\ \frac{dR_{p}}{dt}=b_{1}R_{f}Si_{p}-d_{r}R_{p}\\ \frac{dR_{p}}{dt}=b_{1}R_{f}Si_{m}-d_{r}R_{m}\\ \frac{dD_{e}}{dt}=A_{u}(P+M)+A_{p}((Si_{m}+Sis_{m})P+(Si_{p}+Sis_{p})M)\\ +A_{g}((Si_{m}+Sis_{m})P+(Si_{p}+Sis_{p})M)-G_{d}D_{e}\\ \frac{dSis_{p}}{dt}=0.5G_{d}D_{e}-d_{si}Sis_{p}-b_{1}Sis_{p}R_{f}-A_{p}Sis_{p}M\\ \frac{dSis_{m}}{dt}=0.5G_{d}D_{e}-d_{si}Sis_{m}-b_{1}Sis_{m}R_{f}-A_{p}Sis_{m}P\\ \frac{dR_{sp}}{dt}=b_{1}Sis_{p}R_{f}-d_{r}R_{sp}\\ \frac{dR_{sm}}{dt}=b_{1}Sis_{m}R_{f}-d_{r}R_{sm} \end{array}$$
